# Quick assessment of the economic value of olive mill waste water

**DOI:** 10.1186/s13065-016-0207-7

**Published:** 2016-10-17

**Authors:** Riccardo Delisi, Filippo Saiano, Mario Pagliaro, Rosaria Ciriminna

**Affiliations:** 1 Istituto per lo Studio dei Materiali Nanostrutturati, CNR, via U. La Malfa 153, 90146 Palermo, Italy; 2 Dipartimento di Scienze Agrarie e Forestali, viale delle Scienze, Ed. 4, 90128 Palermo, Italy

**Keywords:** Polyphenols, Hydroxytyrosol, Olive, Tyrosol, Olive mill waste water

## Abstract

**Background:**

Olive biophenols are emerging as a valued class of natural products finding practical application in the food, pharmaceutical, beverage, cosmetic and nutraceutical industries due to their powerful biological activity which includes antioxidant and antimicrobial properties. Olive mill waste water (OMWW), a by-product in olive oil manufacturing, is rich in biophenols such as hydroxytyrosol and tyrosol. The amount of biophenols depends on the cultivar, the geographical area of cultivation, and the seasonal conditions. The goal of this study was to develop a straightforward method to assess the economic value of OMWW via quantification of hydroxytyrosol and tyrosol.

**Results:**

The amount of hydroxytyrosol and tyrosol phenolic compounds in the OMWW from four different cultivars grown in four different regions of Sicily was analyzed using liquid–liquid and solid–liquid analytical protocols developed *ad hoc*. Results showed significant differences amongst the different cultivars and their geographical origin. In all samples, the concentration of hydroxytyrosol was generally from 2 to 10 times higher than that of tyrosol. In general, the liquid–liquid extraction protocol gave higher amounts of extracted biophenols. The cultivar *Cerasuola* had the highest amount of both hydroxytyrosol and tyrosol. The cultivar *Nocellara Etnea* had the lowest content of both biophenols.

**Conclusions:**

A quick method to assess the economic value of olive mill waste water via quantification of hydroxytyrosol and tyrosol in olive phenolic enriched extracts is now available.

## Background

Every year more than 30 million m^3^ of olive mill waste water (OMWW), a mildly acidic red–black emulsion containing 85–92 % water (originating from the olive, the added water required for washing the fruit, and for the centrifugation process) are obtained across the world during the 2 to 3 months of olive oil production, posing one of the biggest environmental problems of today’s agriculture in Mediterranean countries, where 95 % of the world’s olive oil is produced [1]. The average chemical oxygen demand value of OMWW is between 80 and 200 g/L, namely up to 200 times higher than that of domestic effluent and equivalent to the polluting load generated by 22 million people [2]. In general, only 2 % of the total phenolic content of the milled olive fruit goes to the oil phase, while most partitions between the liquid OMWW (≈53 %) and solid pomace (≈45 %) [3].

Biophenols are powerful antimicrobials, and the large phenolics concentration (0.1–18 g/L) in OMWW inhibits both aerobic and anaerobic digestion processes which might turn this waste into irrigation water [4], as well as plant growth in soils in which it was traditionally discharged [5].

On the other hand, olive phenols extracted from OMWW (and from olive leafs) are increasingly commercialized for nutraceutical, dietetic and cosmetic applications due to their exceptional antioxidant and anti-inflammatory properties [6]. The health benefits of olive oil mostly depend on biophenols [7], or polyphenols as they are generally indicated in the scientific literature even though none of these compounds bears two phenolic rings in the molecule [8].

Several epidemiological studies have correlated the low incidence of coronary heart disease, atherosclerosis, and some types of cancer with olive oil consumption in the Mediterranean diet largely practiced in Greece, southern Italy, and Spain [9]. Clinical and biochemical studies are ongoing to evaluate their performance in the treatment of serious neurodegenerative illnesses [10].

In late 2011, the European Food Safety Authority (EFSA) approved an health claim on olive oil phenols, reading as follows: “Olive oil phenols contribute to the protection of blood lipids from oxidative stress” [11]. The claim may be used only for olive oil containing at least 5 mg of hydroxytyrosol (and its derivatives oleuropein and tyrosol) per 20 g of olive oil (with the bottle label informing the consumer that the beneficial effect is obtained with a daily intake of 20 g of olive oil).

To date, more than 50 bio-phenols and related compounds have been identified in olive mill waste. Tyrosol (2-(4-hydroxyphenyl)ethanol), hydroxytyrosol (HT), and their derivative oleuropein are the most abundant (between 60 and 80 % of the total phenolic compounds), depending on the olive cultivar and the geographical origin [12]. Hydroxytyrosol (2-(3,4-dihydroxyphenyl)ethanol), in particular, is characterized by one of the highest antioxidant activities amongst natural and synthetic antioxidant molecules [13], showing cardio-protective and cancer-preventing activity thanks to its powerful inhibition activity of the nuclear factor kappa β, namely the central component of inflammation in chronic inflammatory diseases [14].

The economic value of hydroxytyrosol and tyrosol is very high. For example, as of May 2016 a renowned global chemical supplier was marketing 25 mg of HT (98 % purity) at 225 EUR, and the same amount of tyrosol (same purity) at 303.50 EUR [15]. In this study we describe a methodology to quickly assess the economic value of OMWW samples by determining the amount of tyrosol and hydroxytyrosol.

## Methods

Hydroxytyrosol and tyrosol were purchased from Extrasyntese (Genay Cedex, France). Methanol, *n*-hexane, ethyl acetate, and acetonitrile were obtained from VWR (Milan, Italy). All solvents used were of analytical grade. Four different OMWW samples (5 L) were collected from four different continuous three-phase olive processing mills located in southern and western Sicily (Sciacca and Suvarelli, respectively) and eastern Sicily (Mount Etna) immediately after milling on October 2015. In detail, OMWW samples were obtained from milling *Cerasuola* (from Sciacca mill), *Biancolilla* (Suvarelli), *Tonda Iblea* (Mount Etna, 1000 m above sea level), and *Nocellara Etnea* (Mount Etna, 200 m above sea level) olive cultivars. Olives were in each case grown according to organic (pesticide-free) farming protocols. To avoid decomposition all samples were stored at −20 °C until use. No stabilizing agents were added to avoid chemical alteration of the crude matrices.

### Extraction of OMWW

All OMWW samples were subjected to liquid–liquid and solid–liquid solvent extraction.

#### Liquid–liquid extraction

**Fig. 1:**
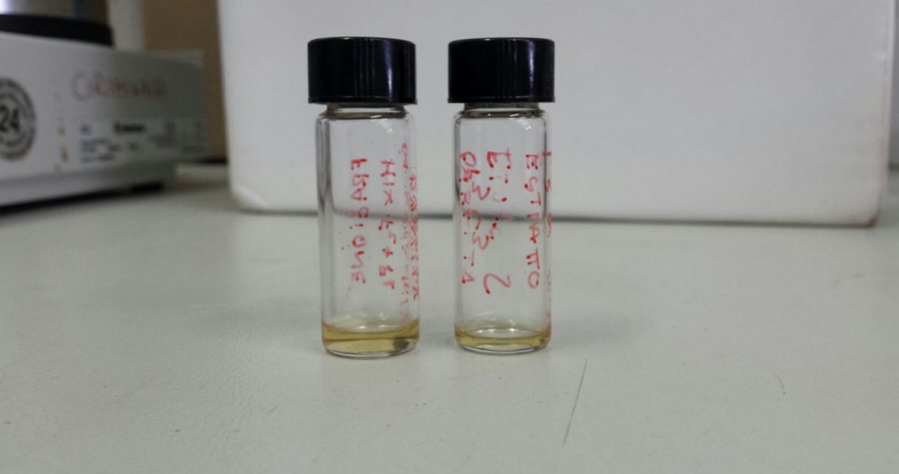
Typical crude extracts obtained via liquid–liquid extraction of OMWW

Typically, one sample of raw OMWW (500 mL) was centrifuged twice at 9000 rpm (Beckman allegra X-22R centrifuge with a fix-angle rotor F0630) in 30 mL vials for 10 min in order to remove pulp and any other suspended solid residues. The resulting water phase was then filtered through Whatman filter paper to get rid of any residual solids. The resulting mildly acidic (pH ≈ 5) green-black aqueous phase was further acidified to pH ≈ 2 using concentrated HCl (2 M). The color of the mixture quickly turned into deep red. The acidified water phase was thus defatted in a separatory funnel using *n*-hexane (3 × 25 mL). The aqueous layer was further extracted with EtOAc (4 × 40 mL) to recover all phenolic compounds, after which the extract was dried over anhydrous Na_2_SO_4_ and evaporated in a rotary evaporator at 40 °C under reduced pressure (180 mbar). A yellow–brown crude oil sample was obtained, depending on the cultivar. To eliminate the resins, each crude sample was separately dissolved in EtOAc and 2 g of silica gel added to the resulting mixture. The solvent was evaporated in a rotary evaporator and the resulting oil adsorbed on silica loaded on a silica gel column (silica gel 60, particle size 0.063–0.200 mm, 70–230 mesh ASTM; 11 g) packed in *n*-hexane. The silica column was then eluted with *n*-hexane (100 mL) to remove the residual non-active apolar components. EtOAc (100 mL) was then added in order to recover the polyphenol fraction. The eluate was evaporated in a rotary evaporator, affording a liquid–liquid polyphenol mixture (LLPM) isolated as yellow-orange oil (Fig. 1).

#### Solid–liquid extraction

In a typical solid–liquid extraction the crude OMWW was first centrifuged and defatted as reported above. Then a sample (50 mL) of the defatted OMWW was lyophilized using a freeze dry system (Freezone 4.5, Labconco corporation) operated at 0.018 mbar and −51 °C. The resulting powder was suspended in MeOH (4 mL) and sonicated for 10 min in an ultrasonic bath (Elma Transsonic 460/H) kept at 30 °C. The methanol extract was filtered through Whatman filter paper, dried over anhydrous Na_2_SO_4_, and evaporated using a rotary evaporator at 40 °C under reduced pressure (150 mbar). Once again, a yellow–orange crude oil was obtained, whose color depended on the cultivar. These oils were separately mixed with silica in a mortar, loaded onto a silica gel septum and purified using the optimized methodology reported above. The four different biophenol extracts obtained from the different cultivars were labelled solid–liquid polyphenol mixtures (SLPM).

### Carbon analysis

Total organic carbon (TOC), total carbon (TC) and inorganic carbon (IC) analyses of each OMWW sample were performed on the defatted and on the centrifuged samples. In detail, a small (500 µL) sample was dissolved in 100 mL of highly pure (milli-Q) water, and the resulting solution filtered through a 0.2 µm Whatman Teflon syringe filter. The resulting solution was analyzed using a Shimadzu TOC-5000A analyzer.

### HPLC–MS analysis

SLPM and LLPM extraction fractions were dissolved, respectively, in 5 and 10 mL of EtOAc. An aliquot (1 μL) of each solution was qualitatively monitored by HPLC and LC–MS by comparison and combination of retention times and mass spectral data (Agilent 6130 Series Quadrupole LC/MS Systems, equipped with G1329A High Performance Autosampler, G1316A Thermostated Column Compartment and G1315D Diode Array Detector). The UV detector was operated at 280 nm. Separation was carried out using an Agilent Eclipse XBD-C18 (4.6 × 150 mm, 5 lm) column maintained at 30 °C. Polyphenol compounds were identified and assessed using a G6120B Single Quadrupole LC/MS system equipped with an electrospray ionization source (ESI). For target compound analysis, a flow injection analysis (FIA) was carried out to determine the fragmentor setting to improve the compound response. The potential chosen was 200 V. Selected ESI work conditions were capillary voltage 5000 V, gas flow rate 13 L/min, gas temperature 300 °C and nebulizer pressure 60 Psi. To obtain the best sensitivity, the quadrupole was used in SIM mode. Optimum separation was achieved with a binary mobile phase gradient at a flow rate 0.5 mL/m. The mobile phase consisted of a binary solvent system using (A) water/formic acid (pH 3.1) and (B) acetonitrile previously degassed.

## Results and discussion

Table 1 shows the pH and carbon content of the OMWW samples obtained from the four different cultivars selected.

**Table 1:** pH and carbon content for the four OMWW samples analyzed

Parameter	*Cerasuola*	*Biancolilla*	*Nocellara etnea*	*Tonda Iblea*
pH	5.11	5.12	5.02	4.98
Inorganic carbon (ppm)	267.8	7.4	13.4	262.6
Total carbon (ppm)	18066	22940	10136	23560
Total organic carbon (ppm)	17798	22920	10122	23280

Table 2 displays the amounts of hydroxytyrosol (HT) and tyrosol (T) found in the LLPM (Fig. 1) and SLPM extracts. Freeze-drying is costly, but the method eliminates stability and storage issues of OMWW whose phenols, during storage, tend to polymerize into high-molecular-weight polymers that are even more difficult to degrade compared to monomer biophenols (1 m^3^ of phytotoxic OMWW with water phytotoxicity mainly attributed to said phenolic compounds [16], in terms of pollution is equivalent to 200 m^3^ of domestic sewage) [17].

**Table 2:** Amounts of hydroxytyrosol (HT) and tyrosol (T) in the LLPM and SLPM extracts

Entry	Cultivar/phenols mixture	HT (mg/L)	T (mg/L)
1	*Biancolilla*/LLPM	65.93	15.80
2	*Biancolilla*/SLPM	83.04	7.81
3	*Cerasuola*/LLPM	125.07	29.64
4	*Cerasuola*/SLPM	42.81	8.89
5	*Tonda Iblea*/LLPM	36.31	3.42
6	*Nocellara Etnea*/LLPM	7.82	4.93
7	*Nocellara Etnea*/SLPM	3.48	0

*LLPM* liquid–liquid polyphenol mixture; *SLPM* liquid–liquid polyphenol mixture

In all samples, the concentration of hydroxytyrosol was generally from 2 to 10 times higher than that of tyrosol. The extracts obtained from *Cerasuola* and *Biancolilla* cultivars had high HT concentration (entries 2 and 3 in Table 2). The highest content of hydroxytyrosol found in the Sicilian OMWW analyzed in this study (entry 3, 125.07 μg mL^−1^) was superior to that found in OMWW samples obtained in Spain (36.0 μg mL^−1^) in 2001 (with both biophenols remarkably absent in French and Portuguese OMWW samples) [6].

Extracts obtained from *Nocellara Etnea* (entries 6 and 7) had a very low amount of HT, and no tyrosol could be isolated via the solid–liquid extraction. The solid–liquid extraction gave better results for the *Biancolilla* OMWW (entries 1 and 2). Best results were obtained via liquid–liquid extraction of the *Cerasuola* OMWW (entry 3). In general, the OMWW samples obtained from *Cerasuola* had concentration of HT about twice than *Biancolilla*, and almost 3.5 and 16 times higher when compared to, respectively, *Tonda Iblea* and *Nocellara Etnea* OMWW samples.

The present results are in agreement with the known variable phenolic content depending on both the cultivar and geographic origin [18]. Biophenols indeed are secondary plant metabolites that act as a defense against ultraviolet radiation, and injury due to oxidation and pathogens, that in response to a very stressing environment or parasitic plant infection (insects, mold, or bacterial) can lead olive trees to increase in the biosynthesis of phenols up to 20 times.

In Sicily, 2015 was a very fruitful year for olives: climate during flowering months was wet, while a summer relatively dry and sunny favored the development of numerous and sane olive fruits. In general, the southern and southeast coast of Sicily receive the least rainfall (less than 50 cm per year), and the northern and northeastern highlands the most (over 100 cm). The phenols concentration in OMWW, thus, is strongly affected by the climate conditions, with the highest phenolics content for (*Cerasuola*) orchards growing in southern Sicily preferably at lower altitudes [19].

## Outlook and conclusions

We have developed a quick methodology to assess the economic value of olive mill waste water based on extraction and analysis of tyrosol and hydroxytyrosol. In both extraction methodologies developed the raw OMWW is first clarified by centrifugation to remove remaining suspended solids. Then either a liquid–liquid (on the clear water phase) or a solid–liquid (on the lyophilized matrices) extraction process is applied to separate the biophenol fraction from other components. Finally, a silica septum is used to remove other hydrophilic resins obtaining a series of biophenol-enriched mixtures (LLPM and SLPM). TOC and HPLC analyses are used to evaluate the total organic content and, in case of sufficient organic content, the amount of hydroxytyrosol and tyrosol. Commercial extraction of olive phenols will be first and foremost applied to those OMWW samples with the highest amount of the latter biophenols, as the economic value of the wastewater streams is directly correlated to the levels of said target compounds.
